# Entropy defect in thermodynamics

**DOI:** 10.1038/s41598-023-36080-w

**Published:** 2023-06-03

**Authors:** George Livadiotis, David J. McComas

**Affiliations:** Department of Astrophysical Sciences, Princeton University, Princeton, NJ 08540 USA

**Keywords:** Fluid dynamics, Astrophysical plasmas

## Abstract

This paper describes the physical foundations of the newly discovered “entropy defect” as a basic concept of thermodynamics. The entropy defect quantifies the change in entropy caused by the order induced in a system through the additional correlations among its constituents when two or more subsystems are assembled. This defect is closely analogous to the mass defect that arises when nuclear particle systems are assembled. The entropy defect determines how the entropy of the system compares to its constituent’s entropies and stands on three fundamental properties: each constituent’s entropy must be (i) separable, (ii) symmetric, and (iii) bounded. We show that these properties provide a solid foundation for the entropy defect and for generalizing thermodynamics to describe systems residing out of the classical thermal equilibrium, both in stationary and nonstationary states. In stationary states, the consequent thermodynamics generalizes the classical framework, which was based on the Boltzmann–Gibbs entropy and Maxwell–Boltzmann canonical distribution of particle velocities, into the respective entropy and canonical distribution associated with kappa distributions. In nonstationary states, the entropy defect similarly acts as a negative feedback, or reduction of the increase of entropy, preventing its unbounded growth toward infinity.

## Introduction

The theory and application of kappa distributions is connected with the existence of correlations among the constituent components of a system. Space plasmas are such systems, with long-range electrostatic interactions coupling the electrons and ions within a Debye sphere^1^. Thus, space plasmas: (1) make a natural laboratory for directly observing various correlated particle distributions, (2) provide observational ground truth for such correlated systems more generally, and (3) are leading to the development of a new paradigm of statistical mechanics and thermodynamics for correlated distributions, which reside in stationary states out of the classical thermal equilibrium.

The probability kappa distribution of a particle having its position and velocity in the infinitesimal intervals $[\vec{r},\vec{r} + d\vec{r}]$ and $[\vec{u},\vec{u} + d\vec{u}]$, respectively, is

1a $$p(\vec{r},\vec{u}\,;\kappa \,,T\,) \propto \,\,\left[ {1 + \frac{1}{\kappa } \cdot \frac{{\varepsilon_{{\text{K}}} (\vec{u}) + \Phi (\vec{r}) - U}}{{k_{{\text{B}}} T}}} \right]^{\, - \kappa - 1} ,$$

where the particle Hamiltonian function sums its kinetic $\varepsilon_{{\text{K}}} (\vec{u})$ and potential $\Phi (\vec{r})$ energies, while *U* is the internal energy per particle. The distribution can be rewritten as

1b $$p(\vec{r},\vec{u}\,;\kappa ,T\,) \propto \,\,\left[ {1 + \frac{1}{{\kappa - \tfrac{1}{2}d}} \cdot \frac{{\varepsilon_{{\text{K}}} (\vec{u}) + \Phi (\vec{r})}}{{k_{{\text{B}}} T}}} \right]^{\, - \kappa - 1} .$$

The total degrees of freedom or dimensionality, *d*, is given by the summation of the kinetic and potential degrees of freedom, $d = d_{{\text{K}}} + d_{\Phi }$, with $d_{{\text{K}}} = \left\langle {\varepsilon_{{\text{K}}} } \right\rangle /(\tfrac{1}{2}k_{{\text{B}}} T)$ and $d_{\Phi } = \left\langle \Phi \right\rangle /(\tfrac{1}{2}k_{{\text{B}}} T)$. The kappa limit values are (i) *κ* → ∞, restoring the classical thermal equilibrium described the MB distributions, and (ii) *κ* → $\tfrac{1}{2}d$ (or *κ* → $\tfrac{1}{2}d_{{\text{K}}}$ in the absence of potential energy, or when $\Phi < < \varepsilon_{{\text{K}}}$), corresponding to the stationary state furthest from the classical thermal equilibrium, also called anti-equilibrium, and described by power-law distributions. Kappa distributions have been employed to describe numerous observations of plasma populations in space plasmas. (For more details on the theory of kappa distributions and its connection to nonextensive statistical mechanics, plasma physics, and thermodynamics, see: the books:^2,3^; the reviews in^4–8^; and, the original applications in:^9–11^.)

Multiple mechanisms that can generate kappa distributions in particle systems. Some examples are: superstatistics^12–17^, effect of shock waves^18^, turbulence^19–21^, effect of pickup ions^22–24^, pump acceleration mechanism^25^, colloidal particles^26^, and polytropic behavior^27,28^. However, there is one unique thermodynamic origin for all of them. The existence of correlations among the particles of a system does not allow the usage of the classical framework based on the Boltzmann^29^—Gibbs^30^ (BG) entropy and Maxwell–Boltzmann (MB)^31^ canonical distribution of particles’ velocity or kinetic energy. Instead, a generalized framework of statistical mechanics and thermodynamics must be used. This framework is based on the kappa distributions and their associated form of entropy^4^, which generalizes the classical Boltzmann–Gibbs formulation and is connected to kappa distributions through the entropy maximization under the canonical ensemble^4,32–35^. The entropy associated with the kappa distributions, or simply, the kappa entropy, coincides with the so-called *q*-entropy, under the transformation of the thermodynamic parameter kappa, *q* = 1 + 1/*κ*. Tsallis (1988) first used this entropic function within the context of statistical mechanics^36^. The statistical framework of kappa distributions also leads to a consistent characterization of temperature for systems residing in stationary states out of thermal equilibrium^4,22,37–40^. The thermodynamic origin of kappa distributions has recently been connected to the new concept of an “entropy defect”^38^.

In previous studies^37^, we used the concept of entropy defect to derive the thermodynamic definitions of temperature and kappa, and determine the entropy of a system of particles with correlations, which generalized the formulation of Sackur-Tetrode entropy^41,42^. We also showed how this concept can be used in entropic equations to determine the impact on the values of kappa for the combining and mixing of two particle populations^24^. Lastly, we showed that the entropy defect explains the thermodynamic origin of kappa distributions^39^.

Here, we show the strict theoretical foundation of the entropy defect and how the basic thermodynamic aspects can be derived from this concept. The entropy defect and the following generalized thermodynamics is suitable for describing particle populations in space plasmas and other systems with correlations among their particles – or constituents, in general – residing in either stationary or nonstationary states. We show that the concept of entropy defect is fundamental and is meaningful even without the necessity of the existence of a thermodynamic stationary state, typically described by the formalism of kappa distributions and their associated entropy. While stationarity is a common and perhaps desirable property, it is not fundamentally required and thus cannot stand as a cornerstone of thermodynamics. In contrast, the entropy defect, which derives from the order induced by physical correlations, can and should always be true; even the classical case of thermodynamics is simply the limiting case of minimized correlations.

The paper is organized as follows. Section "Classical (restricted) versus generalized (non-restricted) Thermodynamics" compares the classical and generalized formulations of thermodynamics: the former is based on the BG entropy, the existence of stationary states described by MB distributions, and the addition rule of entropies; the latter is based on a generalized form of entropy, the existence of stationary states described by kappa distributions, and the summation rule of entropies that includes the classical addition, plus an entropic reduction caused by the existence of correlations. Section "The concept of entropy defect" defines and describes the concept of entropy defect, i.e., its origin and motivation, description for elementary and integrated processes, the definition of its magnitude, which measures the interdependence among the constituents of the system, and the composition of a system with correlation taking into account the entropy defect. Section "Fundamental properties of the entropy defect" introduces the fundamental properties, that is, being separable, symmetric, and (upper) bounded, as a basis of the entropy defect. We show the rationale for these properties first for systems residing in stationary states and then, independently, for systems residing either in stationary or nonstationary states. Collectively, these three properties lead to the specific formulation of the entropy defect. Section "Avoiding infinity" focuses on the property of upper bound and its impact in nonstationary states. Finally, Section "Discussion and Conclusions" summarizes and discusses the conclusions.

## Classical (restricted) versus generalized (non-restricted) thermodynamics

The composition of a system from its smaller constituents is a trivial process and well understood for particle populations in classical thermal equilibrium, which is governed by classical statistical mechanics and thermodynamics. The involved constituent entropies are combined through a simple summation, where the total entropy *S* of a system is the sum of all of the constituent entropies, $S = \sum {S_{i} }$. This additivity rule leads to the extensivity of the system, a macroscopic property characterizing its thermodynamics, i.e., the total entropy *S* of a system composed by *N* constituents of entropy, *S*_*i*_ = *σ*, hence, *S* = *N*∙*σ*.

**Figure 1:**
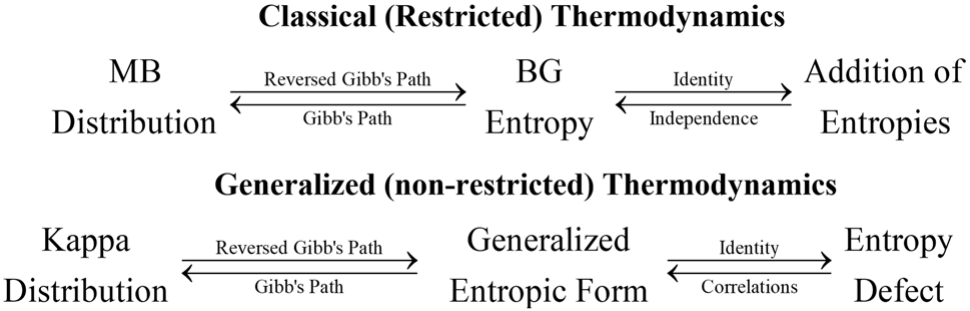
The classical framework of thermodynamics is based on (1) the Boltzmann^29^—Gibbs^30^ entropy, (2) the MB kinetic energy / velocity distribution^31^ that maximizes this entropy under the constraints of the canonical ensemble (e.g.,^4,45^), and (3) the additive property of entropy (e.g.,^46^), where any of these three concepts can lead to the derivation of the other two. The same scheme has been completed in the case of the generalized thermodynamics, which describes systems in stationary states out of the classical thermal equilibrium, including for example, space plasmas.

This entropic summation rule is interwoven with the Boltzmann–Gibbs (BG) formulation of entropy and the Maxwellian velocity distribution that maximizes this entropy within the constraints of the canonical ensemble^4,45,46^. Indeed, this can be explained in two bidirectional arguments (Fig. 1), which connect (i) *canonical distribution with entropy*, and (ii) *entropy with the addition rule*.

(i) Connection of distribution with entropy: Following the Gibb’s path, we maximize the BG entropy $S = - \sum {p\ln p}$ under the constraints of (a) normalization $1 = \sum p$ and (b) internal energy $U = \sum {p \cdot \varepsilon }$, and derive the Maxwell–Boltzmann (MB) energy distribution $p \sim e^{ - \varepsilon /T}$; (the respective velocity distribution can be derived from substituting the kinetic energy). The steps can be reversed: the maximization of entropy, $S = \sum {f(p)}$, leads to the distribution, $f^{\prime}(p) \sim \varepsilon /T + const.$; compared to the MB distribution , we find $f^{\prime}(p) = \ln (1/p) + const.$, leading to $f(p) = - p\ln p$ (taking also into account that in the case of one single possibility, i.e., *p* = 1, we have *S* = 0); that is, the BG entropy.
(ii) Connection* of entropy with entropic addition rule*: Starting from the BG entropy, applied to the two particle systems A, B, and their composed system A + B, noting the respective distributions {*p*} and entropies *S*, we apply (a) the entropic equation $$S = - \sum {p\ln p}$$, and (b) the property of statistical independence, $$p^{{\text{A + B}}} = p^{{\text{A}}} p^{{\text{B}}}$$ or $$\ln p^{{\text{A + B}}} = \ln p^{{\text{A}}} + \ln p^{{\text{B}}}$$. The latter is deduced from the exponential distribution function $$p^{{\text{A + B}}} \sim e^{{ - \varepsilon^{{\text{A + B}}} /T}} = e^{{ - (\varepsilon^{{\text{A}}} + \varepsilon^{{\text{B}}} )/T}} = e^{{ - \varepsilon^{{\text{A}}} /T}} e^{{ - \varepsilon^{{\text{B}}} /T}} \sim p^{{\text{A}}} p^{{\text{B}}}$$ that maximizes this entropy and the energy summation $$\varepsilon^{{\text{A + B}}} = \varepsilon^{{\text{A}}} + \varepsilon^{{\text{B}}}$$. Then, we obtain $$S^{{\text{A + B}}} =$$$$- \sum {p^{{\text{A}}} \ln p^{{\text{A}}} } - \sum {p^{{\text{B}}} \ln p^{{\text{B}}} }$$ $$= S^{{\text{A}}} + S^{{\text{B}}}$$. The reverse process starts from the addition rule and seeks the entropic function that obeys this rule as follows. The addition of two thermodynamically equivalent systems, i.e., those sharing same thermodynamic parameters, produces a similar system, where again its only difference from its constituents is its size. This requires that there be no interactions generating long-range correlations among the particles. Otherwise, the order induced by these correlations would have affected the entropy rule. The absence of correlations means that the two systems are independent, a characteristic of the MB exponential distribution function, and their associated entropies, are given by the BG formulation.

The above argument that connects the addition of entropies to the formulation of entropy is quite informative, because it highlights the natural way of generalizing the classical framework of thermodynamics to describe plasmas and other systems with long-range correlations among their constituents. Hereafter, we describe the system's constituents as its particles, such as space plasma particle populations, but in general they can represent any elementary parcel that constitutes the system.

The existence of particle correlations must affect the entropic addition rule for the physical composition of these systems, and ultimately, points the way to the generalized framework of thermodynamics. The correlations are not a local property that concerns only the closest neighbors of particles, but they rather constitute a global property, characterizing the phase-space of the entire system of particles; (we recall this property, when referring to long-range correlations). If there were no correlations among the two constituent subsystems then, the total entropy of the combined system would have been given by the additivity rule, i.e., $S_{{\text{A + B}}} = S_{{\text{A}}} + S_{{\text{B}}}$. However, when correlations exist among the particles of each of the two subsystems, then further correlations must be developed between the two subsystems during the formation of the combined system.

The “order” generated by the presence of the additional correlations will affect the total entropy of the combined system, which is therefore nonadditive, i.e., $S_{{\text{A + B}}} \ne S_{{\text{A}}} + S_{{\text{B}}}$. The difference between the sum of the subsystems entropies and the total entropy equals a missing amount of entropy, $S_{{\text{A + B}}} = S_{{\text{A}}} + S_{{\text{B}}} - \Delta S$. This defines the “entropy defect”, *S*_D_, which is the decrease of entropy due to the order generated by the presence of additional global correlations in the entire combined system and written as a function of the constituents’ entropies, $\Delta S = S_{{\text{D}}} (S_{{\text{A}}} ,S_{{\text{B}}} )$^37,39^. Anti-correlation of entropy with order is a fundamental property of physical entropy and should be required for good definitions of entropies more broadly. Furthermore, similar to the Shannon’s entropy case^47^, where one can use the asymptotic equipartition property to show that this highly-ordered configuration corresponds to entropy maximization, the kappa entropy is maximized at asymptotic equipartition. In particular, for *M* number of states and equidistribution $p_{i} = 1/M$ the kappa entropy becomes $S_{0} \equiv \kappa \cdot (1 - M^{{ - \tfrac{1}{\kappa }}} )$, but adding a small (meanless) fluctuation from the equidistribution, $p_{i} = 1/M + (\delta_{i} - \overline{\delta })$, the entropy becomes $S_{\delta } = S_{0} - \Delta S$ with $\Delta S \approx \tfrac{1}{2}(1 + \tfrac{1}{\kappa })(M-1)M^{1 - 1/\kappa } \sigma_{\delta }^{2}$.

Classical thermodynamics considers entropies to be additive, leading to the entropy additivity rule among the constituent subsystems A and B, $S_{{\text{A + B}}} = S_{{\text{A}}} + S_{{\text{B}}}$. Indeed, the maximization of the total entropy under the constraint of fixed total energy leads to a special stationary state called classical thermal equilibrium. However, this summation restriction comes simply from the desire for particle systems to be macroscopically extensive, that is, for the total entropy to be proportional to the size of the system. Nevertheless, this restriction is not required, and in fact does not exist for many physical systems. For instance, space plasmas are characterizing by local correlations, where the Debye length defines the radius of a spherical cluster of $N_{{\text{D}}}$ correlated particles, while more distant correlation clusters (Debye spheres) are independent (or “uncorrelated”) due to Debye shielding. Here, the total entropy can be written as a summation over all the $N/N_{{\text{D}}}$ independent clusters, $S_{{{\text{total}}}} = (N/N_{{\text{D}}} ) \cdot S_{{\text{D}}} (N_{{\text{D}}} )$, where $S_{{\text{D}}} (N_{{\text{D}}} )$ denotes the entropy of $N_{{\text{D}}}$ particles characterized by correlations.

If we maximize the total entropy with no assumptions about the rule for the entropy partition (i.e., neither the strict additivity nor other functional restrictions), then, the requirement of a stationary state, called generalized thermal equilibrium, is still possible, but only if there is a reduction in entropy^34,38^. That is, the total entropy can be generally expressed by the classical term of entropy sum, followed by a term that lowers the additive summation of the constituent entropies.

The respective scheme of the two bidirectional arguments in Fig. 1, connecting (i) *canonical distribution with entropy*, and (ii) *entropy with the addition rule*, is in the case of kappa distributions and their associated entropy, as follows:

(i) *Connection of distribution with entropy*: Following the Gibb’s path, we maximize the kappa entropy $S = \kappa (1 - \sum {p^{1 + 1/\kappa } } )$ under the constraints of (a) normalization $1 = \sum p$ and (b) internal energy $0 = \sum {p^{1 + 1/\kappa } \cdot (\varepsilon - U)}$ , and derive the kappa energy distribution $p\sim[1 + \tfrac{1}{\kappa }(\varepsilon - U)/T]^{ - \kappa }$; (the expectation values are determined through the probability $p^{1 + 1/\kappa } /\sum {p^{1 + 1/\kappa } }$, called “escort”^4,48^). Again, the steps can be reversed: the function *f*(*p*) involved in entropy, $S = \sum {f(p)}$, leads to the distribution $p\sim[g(p) + \tfrac{1}{\kappa }(\varepsilon - U)/T]^{ - \kappa }$, with $g(p) \equiv - f^{\prime}(p)p^{{ - \tfrac{1}{\kappa }}} /(\kappa + 1)$; compared to the kappa distribution we find $g(p) = 1$, that gives $f^{\prime}(p) = - (\kappa + 1)p^{{\tfrac{1}{\kappa }}}$, leading to $f(p) = - \kappa p^{{1 + \tfrac{1}{\kappa }}} + const.$, or $f(p) = p \cdot \kappa \cdot (1 - p^{{\tfrac{1}{\kappa }}} )$ (given that *S* = 0 for *p* = 1); that is the kappa related entropy, also named after Havrda/Charvát/Daróczy/Tsallis^36,43,44^. In particular, given the probability distribution {*p*}, the statistical definition of this entropy, is formulated by
  2a $$S_{\kappa } \left( {\{ p\} } \right) = \kappa \cdot \left( {1 - \sum p^{{1 + \tfrac{1}{\kappa }}} } \right) = \left( {1 - \sum p^{q} } \right)/(q - 1),$$
  expressed in terms of the kappa *κ* parameter (mostly known in space science community), or equivalently, the *q*-index (mostly used in the community of nonextensive statistical mechanics) ^4^:
  2b $$q = 1 + 1/\kappa \Leftrightarrow \kappa = 1/(q - 1)$$.
  (2b)The BG entropy is recovered for *κ*→∞ or *q*→1,
  2c $$S_{{{\text{BG}}}} = - \sum {p\ln p} .$$

(ii) *Connection of entropy with a generalized addition rule*: Starting from the entropic functional $1 - \tfrac{1}{\kappa }S = \sum {p^{{1 + \tfrac{1}{\kappa }}} }$, applied again to the two independent particle systems A, B, and their composed system A + B, we find $\sum {(p^{{\text{A + B}}} )^{{1 + \tfrac{1}{\kappa }}} } = \sum {(p^{{\text{A}}} )^{{1 + \tfrac{1}{\kappa }}} } \cdot \sum {(p^{{\text{B}}} )^{{1 + \tfrac{1}{\kappa }}} }$, or $1 - \tfrac{1}{\kappa }S^{{\text{A + B}}} = (1 - \tfrac{1}{\kappa }S^{{\text{A}}} ) \cdot (1 - \tfrac{1}{\kappa }S^{{\text{B}}} )$, leading to $S_{{\text{A + B}}} = S_{{\text{A}}} + S_{{\text{B}}} - \tfrac{1}{\kappa } \cdot S_{{\text{A}}} \cdot S_{{\text{B}}}$. The reverse process starts from this generalized addition rule and seeks the entropic function that obeys this rule; this has been already shown in several earlier publications (e.g. ^38,39^,).

The entropy associated with the kappa distributions, or Havrda/Charvát/Daróczy/Tsallis entropy, has been shown to be consistent with thermodynamics. Nevertheless, beyond this generalized form, there are certainly many other entropic measures with physical connections, important in information theory, and applicable to data and timeseries analysis. Some examples^49^ are: (1) Rényi entropy, used in a different physical context, such as fractal dimension analysis; (2) Kaniadakis entropy, having a strong connection with the formalism of special relativity, and specifically to have entropy follow the same nonadditivity rule as velocity; (note that sometimes this is called K-entropy and should not be confused with the kappa entropy related to kappa distributions); (3) Abe entropy, inspired by the theory of quantum groups, in order to exhibit invariance under transformation of the entropic parameter with its inverse; and 4) Sharma-Mittal entropy, which constitutes the most generalized two parametrical entropic form. It has to be noted though that Rényi entropy is additive, while these other entropic forms cannot be expressed in terms of the entropies of their subsystems (non-composable forms). The specific addition rule is characteristic of the entropic form; for example, entropy additivity is connected with BG or Rényi entropies, while the nonadditivity rule introduced with the entropy defect in this study is related to the Havrda/Charvát/Daróczy/Tsallis entropy. In the future, it will be interesting to investigate the families of entropic forms by their certain nonadditivity rules.

The entropy defect provides a natural generalization of thermodynamics, which can be shown by following similar paths for classical and generalized thermodynamics, as summarized in Fig. 1. The well-known formulation of kappa distributions (e.g.^2,4–7^,) is associated with a generalized form of Havrda/Charvát/Daróczy/Tsallis entropy (gradually developed by^36,43,44^). The entropy maximization (Gibb’s path^30^) leads to stationary states described by kappa distributions^4^, while the reversed path starts from the existence of kappa distributions to find the kappa entropic form (e.g.^45,46^,). (We note that the axiomatic definition of entropy maximization^50^ is consistent^51^ with the kappa or Havrda/Charvát/Daróczy/Tsallis entropy). The characteristic nonadditive rule of the kappa entropy (e.g.,^8^)was first derived on a physical basis through the concept of entropy defect^37^, and then, it was showed that the entropy defect can lead to the generalized, kappa associated entropy^39^. Therefore, the newly developed physical concept of entropy defect should not be confused with what was simply a mathematical derivation of entropy reduction that characterizes nonextensive entropies.

## The concept of entropy defect

### Origin and motivation

Two systems A and B, originally independent of each other, are allowed to interact and mix to compose the system A + B. If no correlations were developed between A and B during this process, then, the entropy of the whole system would have been given by the sum of the entropies of the constituents, i.e.,

3a $$S_{{\text{A + B}}} = S_{{\text{A}}} + S_{{\text{B}}} .$$

However, the existence of correlations between the A and B subsystems—interdependence—adds order to the whole system and thus its total entropy decreases, $S_{{\text{A + B}}} = S_{{\text{A}}} + S_{{\text{B}}} - \Delta S$. This is formulated through the entropy defect, $\Delta S = S_{{\text{D}}} (S_{{\text{A}}} ,S_{{\text{B}}} )$, which is the entropy missing from the total when compared to the summed entropies of the constitutes. Namely:

3b $$S_{{\text{A + B}}} = S_{{\text{A}}} + S_{{\text{B}}} - S_{{\text{D}}} (S_{{\text{A}}} ,S_{{\text{B}}} ),$$

where the entropy defect is generally depended on the involved entropy components.

Therefore, we define the entropy defect as:

4a $$S_{{\text{D}}} \equiv S_{{\text{A}}} + S_{{\text{B}}} - S_{{\text{A + B}}} ,$$

or, in the general combination of an arbitrary number of subsystems, A_1_, A_2_, A_3_ …,

4b $$S_{{\text{D}}} \equiv \sum\limits_{i} {S_{{{\text{A}}_{i} }} } - S_{{\sum\limits_{i} {{\text{A}}_{i} } }} .$$

### Entropy defect for elementary and integrated processes

We distinguish between elementary and integrated processes, as follows. Elementary processes involve single reversible reactions and an isentropic mixing of subsystems A and B, where the entropy defect is used from Eq. (3b). Integrated processes involve a series of iterated elementary processes, where $S_{{\text{A}}} \to S_{n}$, $S_{{\text{B}}} \to \sigma$, $S_{{\text{A + B}}} \to S_{n + 1}$, constructing the difference equation, for *n* = 0,1,…,*N*,

5 $$S_{n + 1} = S_{n} + \sigma - S_{{\text{D}}} (S_{n} ,\sigma ).$$

Then, the total entropy defect sums the defects from all of the involved elementary processes

6 $$S_{N} = S_{0} + N\sigma - S_{{{\text{D,tot}}}} ,S_{{{\text{D,tot}}}} = \sum\limits_{{k = 0}}^{{N - 1}} {S_{{\text{D}}} (S_{k} ,\sigma )} .$$

Furthermore, the elementary entropy defect can be described through elementary processes. For a given elementary process, the entropy defect depends separately on the constituent’s entropies, $S_{{\text{D}}} \propto G(S_{{\text{A}}} ,S_{{\text{B}}} )$, where the proportionality constant quantifies the magnitude of the defect and is denoted with 1/*κ*,

7a $$S_{{\text{D}}} (S_{{\text{A}}} ,S_{{\text{B}}} ) = \frac{1}{\kappa } \cdot G(S_{{\text{A}}} ,S_{{\text{B}}} ).$$

In Sect. Fundamental properties of the entropy defect", we show that the involved function is simply the product of the constituent entropies, $G(S_{{\text{A}}} ,S_{{\text{B}}} ) = S_{{\text{A}}} \cdot S_{{\text{B}}} ,$ i.e.,

7b $$S_{{\text{D}}} (S_{{\text{A}}} ,S_{{\text{B}}} ) = \frac{1}{\kappa } \cdot S_{{\text{A}}} \cdot S_{{\text{B}}} .$$

Hence, Eq. (3b) becomes

8 $$S_{{\text{A + B}}} = S_{{\text{A}}} + S_{{\text{B}}} - \tfrac{1}{\kappa } \cdot S_{{\text{A}}} \cdot S_{{\text{B}}} .$$

### Magnitude of entropy defect: interdependence

The origin of an entropy defect is based on the particle systems’ interdependence (or interconnectedness, that is, the state of physical systems with a significant number of constituents dependent on each other or being connected with each other), quantifying the correlations among their particles, or constituents, in general. Interdependence induces order in the involved systems and reduces their entropy, thus producing the entropy defect. The interdependence is measured through the magnitude 1/*κ* of the entropy defect, defined as

9 $$\frac{1}{\kappa } \equiv \frac{1}{S} \cdot \left( {\frac{{\partial S_{{\text{D}}} }}{{\partial S_{\infty } }}} \right),$$

where *S* is the entropy of the system with correlations among its constituents (e.g., its particle velocities), while *S*_∞_ denotes the entropy of this system if there were absolutely no correlations among them. This equation constitutes the thermodynamic definition of kappa. Systems residing in stationary states have a canonical distribution function and temperature, with kappa representing the well-known kappa that labels and parameterizes the kappa distributions^4^. In addition, the kappa can be connected to a kinetic definition via the correlation coefficient between the particle kinetic energies^52,53^, with the kappa and temperature forming two intensive, and independent, thermodynamic parameters.

The entropy defect can be thought of a negative feedback on the increase of entropy. Namely, the addition of the entropy amount of ΔS_∞_ (a sub-collection of independent constituents) into the system’s entropy *S*, is (partially) defied by the entropy defect ΔS_D_.; thus, $\Delta S \ne \Delta S_{\infty }$, but instead, $\Delta S < \Delta S_{\infty }$ and $\Delta S = \Delta S_{\infty } - \Delta S_{{\text{D}}}$. Then, the magnitude of interdependence or of the entropy defect is the ratio of these quantities, $\Delta S_{{\text{D}}} /\Delta S_{\infty }$, normalized by the value of entropy *S*, $1/\kappa \sim(\tfrac{1}{S}\Delta S_{{\text{D}}} )/\Delta S_{\infty }$. Both entropic amounts can be arbitrarily small, even infinitesimal, leading to Eq. (9).

We observe that the kappa has the following extreme values. First, we write again the definition as $\kappa \sim\Delta S_{\infty } /(\tfrac{1}{S}\Delta S_{{\text{D}}} )$ (in units of $k_{{\text{B}}} = 1$). Then, we have:

- *Maximum Kappa* When there is no entropy defect, meaning there are no correlations amongst the particles of the system, then, entropy is additive and returns to classical BG statistics. The kappa value attains its maximum value: $\Delta S_{{\text{D}}} \to 0$, hence, $\kappa \to \infty$.
- *Minimum Kappa* For the highest possible correlations, the entropy defect attains the maximum possible value, that is, the entropy of the whole system, $\Delta S_{{\text{D}}} \sim S$. On the other hand, the extensive entropy of the system is given by the Sackur-Tetrode entropic equation^41,42^,
  10 $$\frac{{S_{\infty } }}{{k_{{\text{B}}} N}} \sim \frac{3}{2} + \ln \left( {\frac{1}{N} \cdot \frac{V}{{L_{{{\text{th}}}}^{3} }}} \right),$$
  where the system’s volume *V* has to be larger than the volume of a cube with side of a thermal wavelength side $$L_{{\text{t}}}$$, i.e., $$V \ge L_{{{\text{th}}}}^{3}$$. The lower value of the extensive per-particle entropy is $$S_{\infty } /(k_{{\text{B}}} N) \sim \tfrac{3}{2}$$. Since $$S_{\infty } /(k_{{\text{B}}} N) \ge \tfrac{3}{2}$$, then, the minimum value of kappa is just $$\tfrac{3}{2}$$; more general, in the *d*_K_-dimensional case, we have $$S_{\infty } /(k_{{\text{B}}} N) \ge$$$$\tfrac{1}{2}d_{{\text{K}}}$$, hence, $$\tfrac{1}{2}d_{{\text{K}}}$$ becomes the minimum kappa.

This is exactly the range of kappa values, $\tfrac{1}{2}d_{{\text{K}}} < \kappa < \infty$, found for kappa distributions that characterize various stationary states (e.g.,^22,52–54^). Remarkably, here we see that this range also arises from the fundamental definition of kappa given in Eq. (9), based on the entropy defect, and does not require that particle populations even be in kappa distributions.

A measurement of kappa via first principles is equivalent to measuring the correlation of energies between two particles (*i,j*), which provides the kinetic definition of kappa: $\tfrac{1}{\kappa } = \tfrac{2}{{d_{{\text{K}}} }} \cdot \rho$, $\rho = \, < \varepsilon_{i} \varepsilon_{j} > / < \varepsilon_{{}}^{2} >$, (because $< \varepsilon_{i}^{2} > \,\sim\, < \varepsilon_{j}^{2} > \, \equiv \, < \varepsilon_{{}}^{2} >$). This was shown using the formalism of kappa distributions^52^. Now, let the smallest, elementary, amount of entropy, $\sigma$, enter the system, which according to Eq. (10) equals $\tfrac{1}{2}d_{{\text{K}}}$. The corresponding percentage of entropy defect, $(\Delta S_{{\text{D}}} /S)$, stands for the correlation among the entropies, $\rho = \, < \varepsilon_{i} \varepsilon_{j} > / < \varepsilon_{{}}^{2} > \,\sim\,(\Delta S_{{\text{D}}} /S)$. Substituting Eq. (9), we find $\tfrac{1}{\kappa } = \tfrac{{(\Delta S_{{\text{D}}} /S)}}{\sigma } = \tfrac{2}{{d_{{\text{K}}} }} \cdot \rho$, and therefore, we end up with the same result for both the kinetic and thermodynamic definitions of kappa.

### Composition of a system with correlations

#### *Entropy of a composed system with correlations, S*_*κ*_

The composition of a system of *N* constituents is a set of iterations, as in Eq. (5), for the entropy defect *S*_D_ given by Eq. (7b), or Eq. (8), that is, the linear difference equation.

$$S_{n + 1} = S_{n} + \sigma - S_{{\text{D}}} (S_{n} ,\sigma )$$, with $$S_{{\text{D}}} (S_{n} ,\sigma ) = \tfrac{1}{\kappa } \cdot S_{n} \cdot \sigma$$ . (11a).or

11b $$S_{n + 1} = S_{n} + \sigma - \tfrac{1}{\kappa } \cdot S_{n} \cdot \sigma .$$

After the summation of the involved elementary processes, we have

12 $$\begin{gathered} 1 - \tfrac{1}{\kappa }S_{N} = (1 - \tfrac{1}{\kappa }S_{N - 1} ) \cdot (1 - \tfrac{1}{\kappa }\sigma ) = (1 - \tfrac{1}{\kappa }S_{N - 2} ) \cdot (1 - \tfrac{1}{\kappa }\sigma )^{2} \hfill \\ \cdots = (1 - \tfrac{1}{\kappa }S_{n} ) \cdot (1 - \tfrac{1}{\kappa }\sigma )^{N - n} = \cdots = (1 - \tfrac{1}{\kappa }S_{0} ) \cdot (1 - \tfrac{1}{\kappa }\sigma )^{N} , \hfill \\ \end{gathered}$$

or

13 $$S_{N} = \kappa \cdot \left[ {1 - (1 - \tfrac{1}{\kappa }S_{0} ) \cdot (1 - \tfrac{1}{\kappa }\sigma )^{N} } \right].$$

Here, *S*_*N*_ denotes the final entropy of the system, initially having entropy *S*_0_, after the sequential incorporation of *n* = 1, 2, …, *N* individual constituents; each constituent has entropy *σ* when isolated, but it adds to the system less entropy, i.e., $\sigma - \tfrac{1}{\kappa } \cdot S_{n} \cdot \sigma$, due to the entropy defect caused by the existence of correlations among all the particles. Therefore, the composition of a system of *N* correlated constituents, which is characterized by the magnitude of the interdependence, 1/*κ*, is given by

14 $$S_{N} = \kappa \cdot \left[ {1 - (1 - \tfrac{1}{\kappa }\sigma )^{N} } \right],$$

where the system is composed without initial components, namely, *S*_0_ = 0.

**Figure 2:**
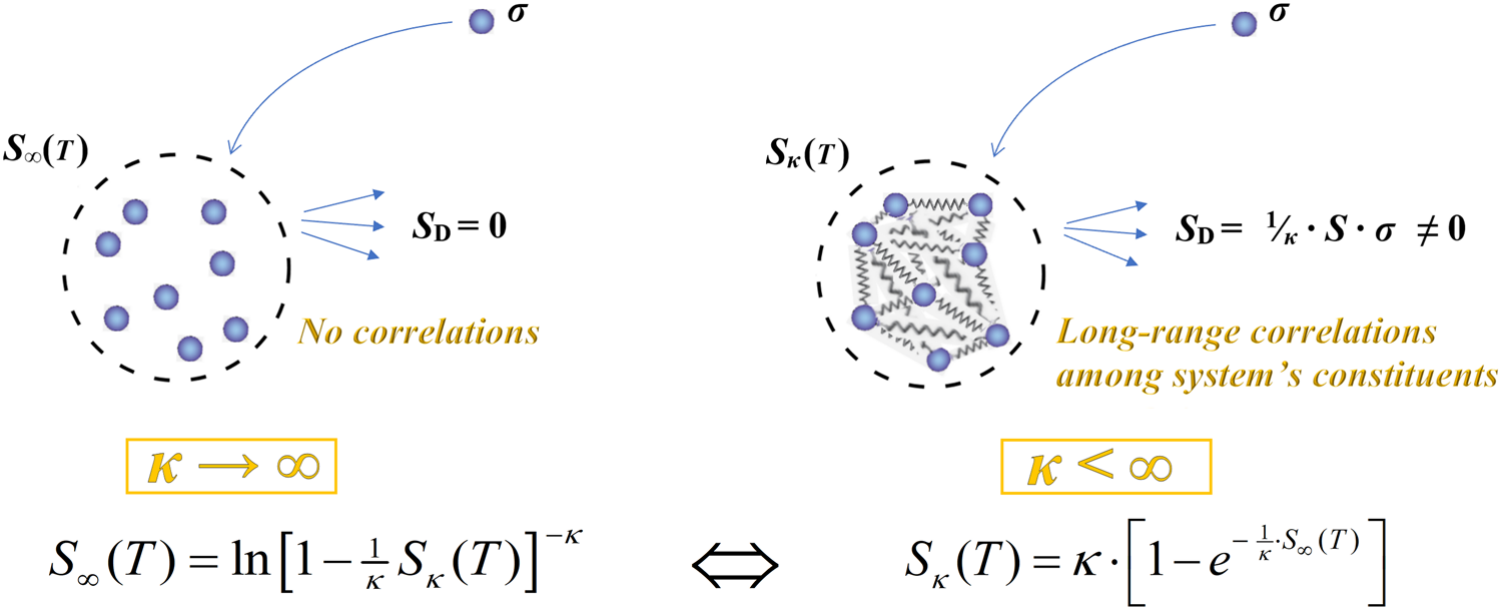
Scheme of the relationship between the entropy of a system, *S*_*∞*_, composed by independent constituents (each of entropy *σ*), with the entropy of the system, *S*_*κ*_, that develops correlations among these constituents. The collection of constituents with no additional correlations leads to the thermal formulation of BG entropy, *S*_∞_(*T*), that is for *κ* → ∞, (left), while the development of additional correlations leads to the thermal formulation of kappa entropy, *S*_*κ*_(*T*), that is for a finite value of *κ* that provides a measure of the correlations. The knowledge of the formulation of any of these two leads to the other.

Next, we focus on comparing the entropy of a system of *N* correlated constituents (characterized by a magnitude 1/*κ*), *S*_*κ*_, as opposed to the entropy *S*_∞_ of a system of the similar size (*N* constituents) but with no correlations (*κ* → ∞). Hence,

15 $$S_{\kappa } = \kappa \cdot \left[ {1 - (1 - \tfrac{1}{\kappa }\sigma )^{N} } \right]\,\,\underset{\kappa < \infty }{\overset{\kappa \to \,\infty }{\rightleftarrows}}\,\,S_{\infty } = N \cdot \sigma ,$$

(also shown in the schemes of Fig. 2). It is useful, to have *S*_*κ*_ expressed directly in terms of *S*_∞_, i.e.,

16 $$S_{\kappa } = \kappa \cdot \left[ {1 - (1 - \tfrac{1}{\kappa N}S_{\infty } )^{N} } \right]\,\,\underset{\kappa < \infty }{\overset{\kappa \to \,\infty }{\rightleftarrows}}\,\,S_{\infty } .$$

Note that for a large number of correlated constituents, this is simplified to

17 $$S_{\kappa } = \kappa \cdot \left[ {1 - e^{{ - \tfrac{1}{\kappa }\ln (1 - \tfrac{1}{\kappa \,N}S_{\infty } )^{ - \kappa \,N} }} } \right]\mathop{\longrightarrow}\limits^{N > > 1}\kappa \cdot (1 - e^{{ - \tfrac{1}{\kappa }S_{\infty } }} ),$$

i.e., Eq. (16) is simplified to

18 $$S_{\kappa } = \kappa \cdot (1 - e^{{ - \tfrac{1}{\kappa }S_{\infty } }} )\,\,\underset{\kappa < \infty }{\overset{\kappa \to \,\infty }{\rightleftarrows}}\,\,S_{\infty } ,$$

and it can be reversed to (Fig. 2)

19 $$S_{\kappa } = \kappa \cdot (1 - e^{{ - \tfrac{1}{\kappa }S_{\infty } }} )\,\, \Leftrightarrow \,\,S_{\infty } = \ln \,(1 - \tfrac{1}{\kappa }S_{\kappa } )^{ - \kappa } .$$

The entropy $S_{\infty }$ stands for the extensive collection of the *N* constituents, each of entropy *σ*, namely, the system of *N* constituents is composed as if there were no correlations. Therefore, by construction, the entropy $S_{\infty }$ is characterized by no correlations or *κ* → ∞. There is no kappa dependence on this entropy, $S_{\infty } \ne S_{\infty } (\kappa )$, hence, all the kappa dependence of the actual entropy *S*_*κ*_ is explicitly given, as shown in Eq. (19). In addition, the temperature dependence on the actual entropy $S_{\kappa } (T)$ is originated from the temperature dependence on the no-correlation entropy $S_{\infty } (T)$, namely, $S_{\kappa } (T) = \kappa \cdot [1 - e^{{ - \tfrac{1}{\kappa } \cdot S_{\infty } (T)}} ]\,\,$. Therefore, knowing the exact formulation of $S_{\infty } (T)$, we can determine $S_{\kappa } (T)$, and vice versa. (See Sect. 5.3).

#### Consistency with the thermodynamic definition of temperature

The existence of stationary states is possible only if the entropy partition can be formulated as:

20a $$H(S_{{\text{A + B}}} ) = H(S_{{\text{A}}} ) + H(S_{{\text{B}}} ) - \tfrac{1}{\kappa }H(S_{{\text{A}}} ) \cdot H(S_{{\text{B}}} ),$$

where the entropy partition function *H* is a positive and monotonically increasing function, and is associated with the thermodynamic definition of temperature:

20b $$\frac{{H^{\prime}(S)}}{{1 - \tfrac{1}{\kappa }H(S)}} \cdot \frac{\partial S}{{\partial U}} \equiv \frac{1}{T}.$$

(Details of this derivation can be found in:^38,40^.) Next, the last equation can be written as

21 $$\frac{{\partial \ln [1 - \tfrac{1}{\kappa }H(S)]^{ - \kappa } }}{\partial U} \equiv \frac{1}{T}.$$

The temperature is independent of the kappa value, thus it would be the same for the classical case of *κ* → ∞ (extensive entropy and no correlations), where the thermodynamic definition is given by

22 $$\frac{{\partial S_{\infty } }}{\partial U} \equiv \frac{1}{T}.$$

Hence, by comparing Eqs.(21, 22), we obtain

23 $$S_{\infty } = \ln [1 - \tfrac{1}{\kappa }H(S)]^{ - \kappa } .$$

At the limit of *κ* → ∞, Eq. (23) leads to $S_{\infty } = \mathop {\lim }\limits_{\kappa \to \infty } H(S_{\infty } )$, hence, we obtain that, in the case where the partition function is independent of kappa, it is just given by the identity function, $H(S) = S$ (similar to the work of^38^). Then, Eq. (20) coincides with the entropy defect, shown earlier in Eq. (8), that is,

24 $$S_{{\text{A + B}}} = S_{{\text{A}}} + S_{{\text{B}}} - \tfrac{1}{\kappa } \cdot S_{{\text{A}}} \cdot S_{{\text{B}}} , \textrm{and} \; \frac{1}{{1 - \tfrac{1}{\kappa }S}} \cdot \frac{\partial S}{{\partial U}} \equiv \frac{1}{T}$$

with the thermodynamic definition of temperature written also as

25 $$\frac{{\partial S_{\infty } }}{\partial U} \equiv \frac{1}{T},\; \textrm{with} \; S_{\infty } = \ln (1 - \tfrac{1}{\kappa }S_{\kappa } )^{ - \kappa } .$$

(Again, $S = S_{\kappa }$ denotes the actual entropy of the system, having the subscript of kappa).

By default, here $S_{\infty }$ represents the classical BG entropy that depends only on temperature and not on kappa, $S_{\infty } = S_{\infty } (T)$. As shown next, knowing any of $S_{\infty } (T)$ and $S_{\kappa } (T)$, that is, the Sackur-Tetrode expressions in terms of temperature, we can construct the other; namely, rewriting Eq. (19), we have:

26 $$S_{\kappa } (T) = \kappa \cdot \left[ {1 - e^{{ - \,\,\tfrac{1}{\kappa } \cdot S_{\infty } (T)}} } \right] \Leftrightarrow S_{\infty } (T) = \ln \,\left[ {1 - \tfrac{1}{\kappa }S_{\kappa } (T)} \right]^{\, - \kappa } .$$

#### *Consistency between the kappa, S*_*κ*_*(T), and classical BG, S*_*∞*_*(T), Sackur-Tetrode entropies*

The expression of the kappa entropy in terms of temperature has been already derived^4,22,35,37^ and^2^ (Chapters 2 and 5)]. According to these derivations, the statistical expression of entropy is maximized under the constraints of canonical ensemble, and then, the resultant stationary distribution is substituted to the maximized entropy. The derived entropy can be expressed in the following compact way:

27a $$S_{\kappa } (T) = \kappa \cdot \left[ {1 - (T/T_{0} )^{{ - \tfrac{1}{\kappa } \cdot \tfrac{1}{2}d \cdot N}} } \right] = \kappa \cdot \left[ {1 - e^{{ - \tfrac{1}{\kappa } \cdot \tfrac{1}{2}d \cdot N \cdot \ln \,(T/T_{0} )}} } \right],$$

where the thermal constant *T*_0_ constitutes the lowest temperature for the entropy to be positive. (For more details, see also:^37–39^.) This formulation serves as a generalization of the Sackur-Tetrode entropy, that is the BG entropy expressed in terms of temperature, i.e.,

27b $$S_{\infty } (T) = \tfrac{1}{2}d \cdot N \cdot \ln \,(T/T_{0} ),$$

that is, Eq. (10), written in the same compact way using *T*_0_. Then, we note that for *κ* → ∞, Eq. (27a) reduces to Eq. (27b).

In order to show the consistency between $S_{\infty } (T)$ and $S_{\kappa } (T)$, we start from Eq. (27a), that gives $S_{\kappa } (T)$. As mentioned, this was found by substituting the kappa distribution into the kappa entropy. When this is compared to Eq. (26) that connects $S_{\infty } (T)$ and $S_{\kappa } (T)$, we end up with $S_{\infty } (T)$ as given by Eq. (27b). The reverse follows by starting from $S_{\infty } (T)$, given by Eq. (27b), substituting it in Eq. (26), we find $S_{\kappa } (T)$, which is exactly the kappa entropy expressed in terms of temperature, shown in Eq. (27a).

## Fundamental properties of the entropy defect

### General aspects

The entropy defect determines how the entropy of the system partitions into its constituent’s entropies and stands on three fundamental properties. In particular, the participation of each constituent’s entropy in the entropy defect must be (i) separable, (ii) symmetric, and (iii) (upper) bounded. We analyze the physical meaning and mathematical formulation of each property that constitutes the foundations of entropy defect:

(i) *Separable.* The entropy defect relates to the constituent’s entropies through a separable function, i.e., $S_{{\text{D}}} (S_{{\text{A}}} ,S_{{\text{B}}} ) \propto S_{{\text{D}}} (S_{{\text{A}}} ,S_{{\text{B}}} = const.) \times S_{{\text{D}}} (S_{{\text{A}}} = const.,S_{{\text{B}}} )$. Following Eq. (7a), this is written in terms of monotonically increasing functions, i.e.,
  28a $$S_{{\text{D}}} (S_{{\text{A}}} ,S_{{\text{B}}} ) = \tfrac{1}{\kappa } \cdot g(S_{{\text{A}}} ) \cdot h(S_{{\text{B}}} ).$$
(ii) *Symmetric.* The entropy of the total system is symmetric to A and B, $S_{{\text{A + B}}} = S_{{\text{B + A}}}$, and the same holds for the entropy defect,$S_{{\text{D}}} (S_{{\text{A}}} ,S_{{\text{B}}} ) = S_{{\text{D}}} (S_{{\text{B}}} ,S_{{\text{A}}} )$. Since entropy defect is separable and symmetric, then it must be *h* = *g* in Eq. (28a), i.e.,
  28b $$S_{{\text{D}}} (S_{{\text{A}}} ,S_{{\text{B}}} ) = \tfrac{1}{\kappa } \cdot g(S_{{\text{A}}} ) \cdot g(S_{{\text{B}}} ).$$
(iii) *Bounded.* The existence of an entropic upper boundary means that the entropies have an upper limit, i.e., $S \le s_{\max }$.

We note that the first two properties of entropy defect, i.e., separability and symmetry, are familiar to the classical understanding of thermodynamics, which hold in the classical case of entropic additivity, because a zero defect is clearly separable and the addition is symmetric, $S_{{\text{A + B}}} = S_{{\text{A}}} + S_{{\text{B}}} = S_{{\text{B}}} + S_{{\text{A}}} = S_{{\text{B + A}}}$. In contrast, the third property, i.e., the existence of an upper boundary, adds an entirely new element in thermodynamics.

The physical reasoning behind the fundamental properties of the entropy defect can be shown independently for systems residing in stationary states, and more generally, without any assumption of stationarity, namely, for systems residing either in stationary or nonstationary states.

### Foundation of entropy defect for systems residing in stationary states.

As mentioned earlier, stationarity is possible^38,40^, only if entropies partition as:

29 $$H(S_{{\text{A + B}}} ) = H(S_{{\text{A}}} ) + H(S_{{\text{B}}} ) - \tfrac{1}{\kappa }H(S_{{\text{A}}} ) \cdot H(S_{{\text{B}}} ),H(S) > 0,H^{\prime}(S) > 0,$$

Hence, we have:

(i) *Separable.* The third, non-additive, term $H(S_{{\text{A}}} )H(S_{{\text{B}}} )$ is just a product and so obviously separable.
(ii) *Symmetric.* Again, for the third term, we have an algebraic identity $H(S_{{\text{A}}} )H(S_{{\text{B}}} ) = H(S_{{\text{B}}} )H(S_{{\text{A}}} )$.
(iii) *Bounded.* The formulation in Eq. (29) implies the existence of an entropy upper boundary. Indeed, *H* is a positive function of entropy, i.e., for $S_{{\text{A + B}}} \ge 0$, then $H(S_{{\text{A + B}}} ) \ge 0$, leading to $1/H(S_{{\text{A}}} ) + 1/H(S_{{\text{B}}} ) \ge 1/\kappa$. The subsystems A and B are independent before they combine to compose the total system A + B, so the inequality also has to hold independently for each component. If *H*(*S*) has no upper boundary, then its reciprocal can be as small as $1/H(S) \to 0$ (that is, for those values of *S* that $H(S) \to \infty$). However, this could hold for both A and B entropies (since they act independently), and then, the inequality $1/H(S_{{\text{A}}} ) + 1/H(S_{{\text{B}}} ) \ge 1/\kappa$ would have been violated. Therefore, there must be finite upper boundary for *H*(*S*), i.e., $H(S) \le h_{\max }$. If $H(S_{{\text{A}}} )$ attains this upper limit, then, the addition of more entropy would still maintain the total entropy as invariant, i.e., if $H(S_{{\text{A}}} ) = h_{\max }$, then $H(S_{{\text{A + B}}} ) = h_{\max }$. Hence, substituting in Eq. (29), we obtain $H(S_{{\text{B}}} ) \cdot (1 - \tfrac{1}{\kappa }h_{\max } ) = 0$ or $h_{\max } = \kappa$. Finally, the upper bound of the positive and monotonically increasing function *H*(*S*) indicates an upper boundary of the entropy *S*, i.e., $H(S) \le h_{\max }$ or $S \le H^{ - 1} (h_{\max } ) \equiv s_{\max }$.

### Foundation of entropy defect for systems residing either in stationary or nonstationary states.

Here, we show the three properties, in general, without the necessity of Eq. (29), that is, of the system residing in a stationary state.

(i) *Separable.* Originally (before mixing), the constituent subsystems are independent of each other. This fact can be used to justify the separability of the entropy defect, as follows. For different values of any of the two entropic components, let $S_{{\text{A}}} = a_{1}$ and $S_{{\text{A}}} = \,a_{2}$, the respective entropy defect values,$S_{{\text{D}}} (S_{{\text{A}}} = a_{1} ,S_{{\text{B}}} )$ and $S_{{\text{D}}} (S_{{\text{A}}} = a_{2} ,S_{{\text{B}}} )$ should have the same mathematical dependence on *S*_B_, or equivalently, the ratio $S_{{\text{D}}} (S_{{\text{A}}} = a_{1} ,S_{{\text{B}}} )/S_{{\text{D}}} (S_{{\text{A}}} = a_{2} ,S_{{\text{B}}} )$ must be independent of *S*_B_. Rewriting this with two infinitesimally different values, $S_{{\text{A}}} = a$ and $S_{{\text{A}}} = a + da$, the two entropy defect values are $S_{{\text{D}}} (a,S_{{\text{B}}} )$ and $S_{{\text{D}}} (a,S_{{\text{B}}} ) + [\partial S_{{\text{D}}} (S_{{\text{A}}} ,S_{{\text{B}}} )/\partial S_{{\text{A}}} ]_{{S_{{\text{A}}} = a}} \cdot da$, which have the same dependence on *S*_B_. Then, their ratio $1 + [\partial \ln S_{{\text{D}}} (S_{{\text{A}}} ,S_{{\text{B}}} )/\partial S_{{\text{A}}} ]_{{S_{{\text{A}}} = a}} \cdot da$ is independent of *S*_B_, for any *a*; equivalently, by excluding the constants, we have that $\partial \ln S_{{\text{D}}} (S_{{\text{A}}} ,S_{{\text{B}}} )/\partial S_{{\text{A}}}$ is independent of *S*_B_, or $\partial^{2} \ln S_{{\text{D}}} (S_{{\text{A}}} ,S_{{\text{B}}} )/\partial S_{{\text{A}}} \partial S_{{\text{B}}} = 0$. Equation $\partial^{2} \ln f(x,y)/\partial x\partial y = 0$ is a property of the 2D function *f* to be separable in *x* and *y*, so $S_{{\text{D}}} (S_{{\text{A}}} ,S_{{\text{B}}} )$ is separable.
(ii) *Symmetric.* For two constituents A and B composing the total system, it does not make any physical sense that it would matter if A was added to B or B was added to A, or even, which was labeled which, to start with. Therefore, the entropy of the total system is symmetric to A and B, $S_{{\text{A + B}}} = S_{{\text{B + A}}}$, and the same holds for the entropy defect,$S_{{\text{D}}} (S_{{\text{A}}} ,S_{{\text{B}}} ) = S_{{\text{D}}} (S_{{\text{B}}} ,S_{{\text{A}}} )$.
(iii) * Bounded.* Starting from entropy *S*_A_, the addition of another entropy, *S*_B_, including the entropy defect, produces: $S_{{\text{A + B}}} = S_{{\text{A}}} + S_{{\text{B}}} - S_{{\text{D}}}$ The second law of thermodynamics requires, $S_{{\text{A}}} \le S_{{\text{A + B}}}$ or $S_{{\text{D}}} \le S_{{\text{B}}}$. From Eq. (28b), $S_{{\text{D}}} = \tfrac{1}{\kappa } \cdot g(S_{{\text{A}}} ) \cdot g(S_{{\text{B}}} )$, we obtain $S_{{\text{A}}} \le g^{ - 1} [\kappa \cdot S_{{\text{B}}} /g(S_{{\text{B}}} )]$, since *g* is a monotonically increasing function. The inequality ensures that the entropy *S*_A_ has an upper bound.

### Consequences of the bound’s universality on the formulation of the entropy defect

The existence of a universal upper bound leads to the determination of the arbitrary separable function *g* included in the entropy defect, Eq. (28b). Indeed, this can be shown in two independent ways:

- We observe that the derived upper limit, $S_{{\text{A}}} \le g^{ - 1} [\kappa \cdot S_{{\text{B}}} /g(S_{{\text{B}}} )]$, is universal, that is, independent of *S*_B_, only if the separable function *g* is the identity function, $g(S) \propto S$ or $g(S) = S$ (without any loss of generality, since the proportionality constant is absorbed in the notion of kappa). Then, the limit becomes $S_{{\text{A}}} \le \kappa \equiv s_{\max }$.
- The existence of an upper bound leads to a specific form of the function *g* for the entropy defect as shown in (28b). Substituting this equation into the entropy summation rule (3b), we have
  30 $$S_{{\text{A + B}}} = S_{{\text{A}}} + S_{{\text{B}}} - \tfrac{1}{\kappa } \cdot g(S_{{\text{A}}} ) \cdot g(S_{{\text{B}}} )$$

﻿Obviously, setting $S_{{\text{B}}}$ = 0 must lead to $S_{{\text{A + B}}} = S_{{\text{A}}}$, hence, $g(0) = 0$. Moreover, if the entropy of the system A attains its upper boundary, $S_{{\text{A}}} = s_{\max }$, then, the further addition of entropy would lead to the same value, $S_{{\text{A + B}}} = s_{\max }$, and Eq. (30) simplifies to $g(S_{{\text{B}}} ) = const. \cdot S_{{\text{B}}}$, where the proportionality constant equals $\kappa /g(s_{\max } )$; hence, we find:

31 $$S_{{\text{D}}} (S_{{\text{A}}} ,S_{{\text{B}}} ) = \tfrac{1}{\kappa } \cdot S_{{\text{A}}} \cdot S_{{\text{B}}} ,\; \textrm{with} \; s_{\max } = \,\kappa ,$$

and the addition rule becomes

32 $$S_{{\text{A + B}}} = S_{{\text{A}}} + S_{{\text{B}}} - \tfrac{1}{\kappa } \cdot S_{{\text{A}}} \cdot S_{{\text{B}}} .$$

Finally, we note that the upper limit tends to infinity in the classical thermal equilibrium, i.e., $S \le s_{\max } = \kappa \to \infty$, indicating that in the classical case, entropies can constantly increase, tending to infinity, and eventually attain any possible positive value.

## Avoiding infinity

Finite systems cannot attain infinite entropy, but continuous irreversible and spontaneous increases of entropy can eventually lead the total entropy toward nearly infinite values. In the classical picture, continuous mixing of particle systems constantly increases their entropy. For instance, the continuous mixing in space plasmas, which have time scales many orders of magnitude smaller than the age of the universe, should have led to tremendously large entropies. In reality, however, this is not what is observed.

In the classical understanding of thermodynamics, the entropy is allowed to constantly increase toward infinity. However, the newly discovered requirement of an upper bound restricts the continuous increase of entropy, ensuring that there are only finite values of entropy.

For a continuous addition of entropy $\Delta S = \sigma$ in a time-scale of $\Delta t = \tau$, we have the difference equation $S_{n} = f(S_{n - 1} )$ that connects the entropy of the *n*th iteration with the previous, (*n*-1)th entropy. The classical case of unrestricted addition is trivially formulated with $S_{n} = S_{n - 1} + \sigma$, that is, solved to $S_{n} = S_{0} + \sigma \cdot n$. The passing of time measures iterations, i.e., $t = n \cdot \tau$, while the constant entropic rate is $\dot{\sigma } = \sigma /\tau$. The corresponding differential equation is $dS/dt = \dot{\sigma }$, that is, solved as $S_{t} = S_{0} + (\sigma /\tau ) \cdot t$ or $S_{t} = S_{0} + \dot{\sigma } \cdot t$. Clearly, the entropy is not bounded, $S_{t \to \infty } \to \infty$.

Now, we repeat the previous steps, but including the restricted addition in terms of the entropy defect, i.e., $S_{n} = S_{n - 1} + \sigma - \tfrac{1}{\kappa }S_{n - 1} \sigma$, that is, solved to $S_{n} = \kappa \cdot [1 - (1 - \tfrac{1}{\kappa }S_{0} ) \cdot (1 - \tfrac{1}{\kappa }\sigma )^{n} ]$^37^. Then, for the passing of time $t = n \cdot \tau$, we obtain $S_{t} = \kappa \cdot [1 - (1 - \tfrac{1}{\kappa }S_{0} ) \cdot (1 - \tau \tfrac{1}{\kappa }\dot{\sigma })^{t/\tau } ]$; for small timescales *τ* of each iteration this is written as $S_{t} = \kappa \cdot [1 - (1 - \tfrac{1}{\kappa }S_{0} ) \cdot e^{{ - \tfrac{1}{\kappa }\dot{\sigma }t}} ]$ that solves the differential equation $dS/dt = \dot{\sigma } - \tfrac{1}{\kappa }\dot{\sigma }S$. We observe that the entropy is now bounded, $S_{t} \le s_{\max } = \kappa$, tending to this limit, $S_{t \to \infty } \to \kappa$. Then, the entropy is given as a function of time, with (33a) or without (33b) the entropy defect:

33a $$S_{t} = \kappa \cdot [1 - (1 - \tfrac{1}{\kappa }S_{0} ) \cdot e^{{ - \tfrac{1}{\kappa }\dot{\sigma }t}} ],\quad S_{t \to \infty } \to \,\kappa ,$$

33b $$S_{t} = S_{0} + \dot{\sigma } \cdot t,\quad S_{t \to \infty } \to \infty ,$$

**Figure 3:**
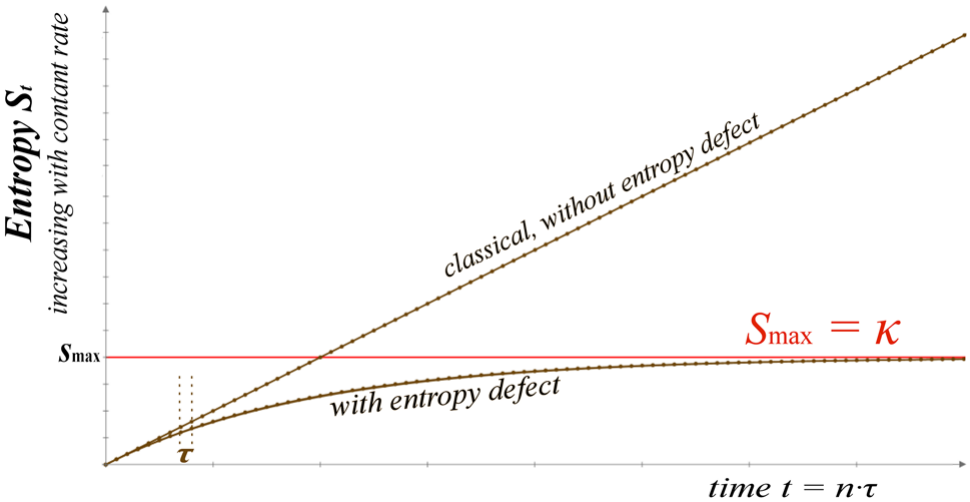
Entropy *S*_*t*_ increasing with time, *t* = *n*∙*τ*, at a constant rate, plotted with and without the entropy defect; *n* counts the iterations, where each iteration has a time scale of *τ*. In the case of no entropy defect, the entropy continuously increases to infinity. In contrast, the entropy defect implies an upper limit *s*_max_ = *κ* that bounds the entropy, and thus the continuous increase of entropy leads to a finite value of entropy, *s*_max_.

The classical case is simply the limiting case of entropy defect, given for *κ* → ∞. Figure 3 plots the entropy in both the cases, where we observe the limit of entropy in the case of entropy defect as opposed to the constantly increasing entropy in the classical case of entropy summation without entropy defect.

## Discussion and conclusions

The paper has provided the strict foundation of the entropy defect. This is analogous to the well-known mass defect, but instead, it describes the decrease of entropy caused by the order induced in the system through the existence of correlations among the system’s particles, or constituents, in general. The entropy defect affects the composition of a system from its constituents and the summation rule expresses the entropy of the system in terms of the constituent entropies. In particular, the existence of correlations between the constituent subsystems—interdependence—adds order to the whole system and thus decreases its total entropy, leading to the entropy defect, a term that reduces the simple summation of the constituent entropies.

We showed that the entropy defect arises from three fundamental properties of the partition of the entropy of a system to its constituents’ entropies. Specifically, each constituent’s entropy must be (i) separable, i.e., the expression of the entropy defect involves separately each term that corresponds to the entropy of each constituent, (ii) symmetric, i.e., the entropy defect is invariant under permutations of any two constituents, and (iii) bounded, i.e., existence of an upper boundary to entropy, restricting it to finite values. We described the physical meaning and mathematical formulation of each property in detail.

The property of acquiring an upper boundary is novel compared with classical BG entropy. According to this property, any entropy has an upper limit given by the kappa value that characterizes the system. The kappa parameter tends to infinity in the case of the classical thermal equilibrium. Therefore, the entropy becomes unbounded exactly at the classical limit. On the other hand, the finiteness of kappa values means there is an upper bound to the entropy values, preventing it from increasing towards infinity. As an example, the case of a system in nonstationary state with constant entropy rate was examined.

The property of boundedness also has more general implications. For example, it can have consequences on the possible scenarios of the universe’s future, especially to the one popularly called “Heat Death”^55^. According to this scenario, in its distant future, the universe will ultimately reach thermal equilibrium. Then, no further work will be possible, resulting in a final heat death of the universe. There have been several suggested “rescuing” scenarios, in which the universe might possibly avoid eternal heat death (e.g.,^56^). Understanding thermodynamics within the context of entropy defect can provide a natural way for “saving” the universe from an ultimate heat death, since its entropy is bounded and cannot continuously increase to infinity.

The formalism of kappa distributions and their associated entropy have their thermodynamic origin in the existence of the entropy defect^39^. This is the cornerstone of the consequent thermodynamics and holds for systems residing either in stationary or nonstationary states. The kappa value is introduced as the magnitude of the entropy defect that measures the interdependence (or interconnectedness) among the constituents of the system, the correlations among the particles. Systems residing in stationary states have a canonical distribution function and temperature, which coincides with that formalism of kappa distributions. Then, the kappa defining the magnitude of entropy defect represents the well-known kappa that labels and parameterizes the kappa distributions^4^. We showed that the kappa has exactly the range of values found for kappa distributions in stationary states^22,52–54^, in general, even for systems residing out of stationary states. We also showed a first principles consistency of kinetic and thermodynamic definitions of kappa and how this can be used to measure its value without having to fit the distribution of particle velocities. An interesting future analysis would examine the various possible ways of measuring the kappa value of a system strictly from the way entropy is partitioning into its constituents.

The concept of entropy defect leads to the thermodynamic origin of kappa distributions, for systems residing in stationary states. The following thermodynamics uniquely generalizes the classical framework based on the Boltzmann–Gibbs entropy and the Maxwell–Boltzmann canonical distribution of particles velocity or kinetic energy. This is suitable for also describing the thermodynamics of systems residing in stationary states out of the classical thermal equilibrium, such as plasma particle populations from laboratory plasmas under extreme conditions (e.g.,^57^) and space plasmas throughout the heliosphere and beyond (e.g.,^2^, and refs. therein).

Kappa distributions have successfully described the particle velocities in a plethora of space plasmas from solar wind and the planetary magnetospheres to the inner heliosheath, and even beyond, to interstellar plasmas^2,4,6,7^. In particular, since launching in 2008, the Interstellar Boundary Explorer (IBEX) mission^58,59^, has been providing remarkable evidence of kappa distributions in the plasma populations at the outer boundaries of the heliosphere. The mission measures energetic neutral atoms (ENAs), which are produced by charge exchange between energetic ions and cold interstellar neutral atoms and subsequently transit back into 1 au. IBEX measures ENA energy-flux spectra over thousands of pixels across the sky, which have been used to remotely determine the thermodynamic parameters of the inner heliosheath^60,61^. The Interstellar Mapping and Acceleration Probe (IMAP,^62^), slated to launch in 2025, will carry these observations to an even higher level, enabling more precise and higher resolution determination of the thermodynamic properties of and processes at work in the outermost regions of our heliosphere.

Beyond just particle distributions and thermodynamics, there has been exponential growth in the number of publications studying and applying the formalism of kappa distributions, their associated entropy, and its “thermodynamics.” Such disciplines include: (i) sociology-sociometry, e.g., internet^63^, urban agglomeration^64^; (ii) linguistics^65^; (iii) economics^66^; (iv) biochemistry^67^ and biology^15^; (v) applied statistics^68^; (vi) nonlinear dynamics^15^; (vii) physical chemistry^69^; (viii) plasma physics^2^; (ix) solid state physics, e.g., spin glass relaxation^70^ and optical lattices^71^, (x) high energy experiments^72^, and many others. The development and understanding of the entropy defect established here and in^37,39^ is similarly valuable in these sorts of systems, where correlations, and interdependence or interconnectedness more broadly, are applicable.

**Figure 4:**
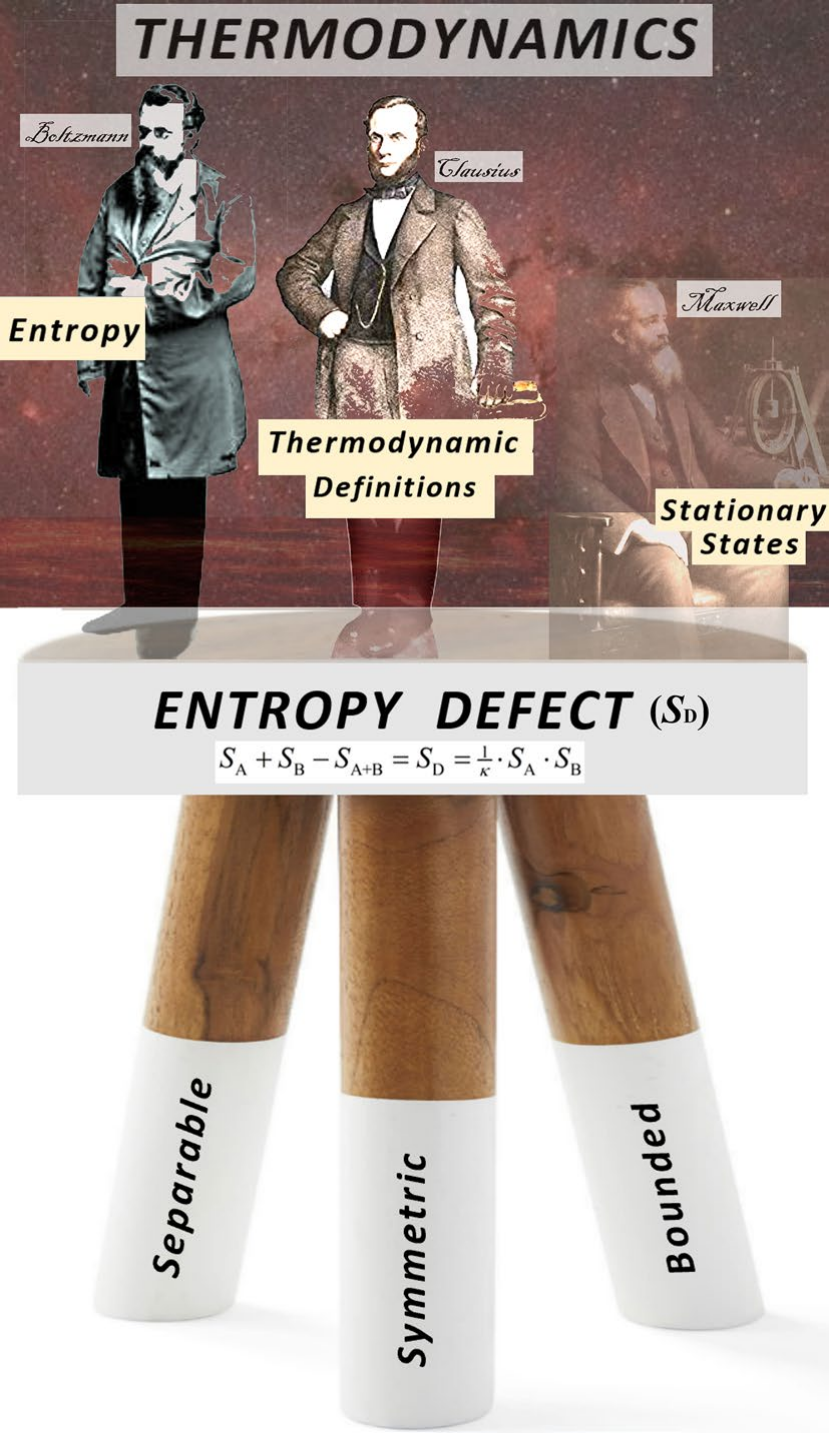
The three foundations of the entropy defect; that is, it must be (i) separable, (ii) symmetric, and (iii) bounded. The entropy defect can lead to the basics of thermodynamics: (i) entropy; (ii) thermodynamic definitions of intensive parameters, and (iii) existence of stationary states and their canonical distributions.

Finally, Fig. 4 summarizes the three foundations of the entropy defect, which stands as the cornerstone for generalizing thermodynamics to describe systems residing out of the classical thermal equilibrium, again, either in stationary or nonstationary states. The entropy defect leads to the generalized thermodynamics (and visa versa). This framework of thermodynamics comprises the following aspects: (i) entropy, which is associated with the formalism of kappa distributions^39^ and coincides with a well-known form of entropy^36,43,44^, generalizing the BG entropy^29,30^; (ii) thermodynamic definitions of the intensive parameters of temperature and kappa^37^, generalizing the classical definition^73^; and (iii) existence of stationary states^38,40^, which are typically described by kappa distributions (e.g.^2,4–7^), generalizing the MB distributions^31^. Thus, it is now straightforward to use the strength and capabilities of thermodynamics consistent with the concept of entropy defect to describe space plasmas and other any other systems characterized by correlations among their constituents.

## Data Availability

All data generated or analysed during this study are included in this published article.
